# Immediate implant replacement with DIEP flap: a single-stage salvage option in failed implant-based breast reconstruction

**DOI:** 10.1186/s12957-018-1387-5

**Published:** 2018-04-17

**Authors:** Miguel De La Parra Marquez, Ricardo Fernandez-Riera, Hector Vela Cardona, Jesus María Rangel Flores

**Affiliations:** 1 Division of Plastic and Reconstructive Surgery, Mexican Institute of Social Security (IMSS), No. 21 Pino Suárez y 15 de Mayo, Av. Hidalgo 2480 pte, col. Obispado. C.p.64060. Cons. 212, Monterrey Nuevo León, Mexico; 2 Department of Plastic and Reconstructive Surgery, Hospital General Dr. Ruben Leñero, Plan de San Luis esq Salvador Díaz Mirón SN, Col. Santo Tomás. Deleg. Miguel Hidalgo. Cp. 11340. CDMX, Mexico City, Mexico

**Keywords:** Tertiary breast reconstruction, Autologous breast reconstruction, Implant failure, Reconstruction failure

## Abstract

**Background:**

Implant-based immediate breast reconstruction after skin-sparing mastectomy has shown a significant improvement in patients’ quality of life, making the procedure steadily more popular year after year. However, this technique has a high morbidity rate, including skin necrosis and implant exposure.

**Methods:**

A retrospective review of a prospectively held database for autologous breast reconstruction in our institution of the last 5 years found eight cases with exposed implants after nipple-sparing mastectomy and immediate reconstruction. A single-stage procedure consisting on implant removal and immediate replacement with a deepithelialized DIEP flap was performed in all cases (10 DIEP flaps).

**Results:**

All flaps were successful. Patients’ mean age was 45 years old. Three patients developed seroma (5, 7, and 14 days after surgery, respectively). No infections were detected in up to 24 months of follow-up.

**Conclusions:**

Nipple-sparing mastectomy with immediate implant-based reconstruction is considered oncologically safe. However, it has a high rate of complications that could require implant removal. Immediate free flap reconstruction is a feasible and safe option to replace the missing volume with low risk of complications that result in a soft and natural-shaped breast.

## Background

Proven improvement in most patients’ quality of life has made the frequency of immediate breast reconstruction after skin-sparing mastectomy to steadily rise in the last years. Women are also increasingly demanding nipple-sparing and skin-sparing procedures from their surgical oncologists [1].

To date, there is no universally accepted criteria for patient selection for skin-sparing mastectomy; however, the most accepted are tumor size less than 3 cm, tumor location greater than 2 cm from the nipple-sparing mastectomy, clinically negative axillary nodes, absence of skin involvement or inflammatory cancer, and clean margins beneath the nipple [2–5].

Traditionally, immediate breast reconstruction with implants requires a two-stage procedure including subpectoral placement of a tissue expander at the time of mastectomy followed by replacement of the tissue expander with the definitive breast implant as a second stage once breast expansion has been achieved. Acellular dermal matrix use for pocket creation and total implant coverage after nipple-sparing mastectomy is booming as a one-stage implant-based reconstruction option and has been widely studied and discussed in medical literature [6–15].

Implant-based reconstruction is associated with risks and complications, which may lead to complete implant loss due to infection, implant exposure, or capsular contracture [16–18], the latter with reported incidence higher than 50% of all implant-based reconstructions [19]. Breast infection or implant exposure increases the rate of failure by 30%, and implant loss has been reported to occur in 4 to 18% of all prosthetic breast reconstructions [16]. Failure of primary or secondary breast reconstruction creates a stressful situation for both patient and surgeon, and decisions should be taken to adjust the strategy and eliminate potential causes of recurrent failure [18].

Hamdi et al. coined the term “tertiary breast reconstruction” for those “redo” reconstructions of cases with unsatisfactory results or failure of previous immediate or delayed procedures [17]; several authors have used that term afterwards [20–23].

The goal of tertiary reconstruction is the complete restoration of the breast after a failed previous reconstruction; it can be achieved with an implant, autologous tissue, or a combination of both. Long-term implant-based reconstruction complications and improvement on microvascular techniques are making patients ask for autologous reconstruction more often [16, 24]. In 1994, Feng et al. reported the use of autogenous tissue for breast reconstruction following implant failure [25]; nowadays, there are several autologous flaps used for this purpose, such as deep inferior epigastric artery perforator flap (DIEP), superior gluteal artery perforator flap (SGAP), transverse musculocutaneous gracilis flap (TMG), superficial inferior epigastric artery perforator flap (SIEA), and many others [15]. These procedures have the advantage of resulting in a breast that responds to weight changes, that has a natural texture, and that eliminates the risk of capsular contracture, but they require certain microsurgical expertise, lead to additional scars in the donor site, and need longer surgeries than their prosthetic-based counterparts [18, 26, 27].

The purpose of this study was to prove the safety and feasibility of the management of implant exposure after nipple-sparing breast mastectomy and implant-based reconstruction with implant removal and immediate replacement with DIEP flap.

## Methods

For a period of 5 years (starting June 2012), we have prospectively held an independent database for patients admitted at our institution for breast reconstruction. We performed a retrospective review of this database looking for all patients referred to the plastic surgery department of our hospital who had previously been managed with nipple-sparing mastectomy and immediate prosthetic-based reconstruction and developed implant exposure. A one-stage procedure with implant removal and immediate replacement with DIEP flap was performed in all cases. Demographic data and complications were studied to assess the safety and feasibility of our single-stage tertiary breast reconstruction technique.

### Operative technique

Preoperative markings are performed with the patient in the standing and supine positions. The superior margin of the flap is shifted slightly above the umbilicus to include periumbilical perforators. Perforators are identified with a handheld 8 MHz Doppler. No additional image studies are routinely performed in our institution.

The procedure is approached by two teams. In the abdomen, DIEP flap is elevated from lateral to medial in a suprafascial plane until adequate perforators are found. The superficial inferior epigastric vein (SIEV) is preserved. The largest perforators are dissected, and the anterior rectus sheath is opened around the perforating vascular bundle, allowing the perforators to be traced to the deep inferior epigastric vessels. Intercostal nerves should be left intact to avoid denervating the muscles medially. The rectus sheath and muscle are separated to allow isolation of the pedicle until desired pedicle length and diameter is obtained.

Simultaneously, the second team proceeds to remove the exposed implant, debride the borders of the skin defect, and wash the pocket with iodine and saline solutions, hypochlorous acid solution also being a good alternative. After pocket irrigation, we perform multiple capsulotomy incisions to attain good compliance of the chest flap. The internal mammary artery and vein are dissected as the recipient vessels of choice; when the area of implant exposure is too close to the inframammary fold, the fourth rib is selected; otherwise, the third rib level is preferred. We approach the vessels by resecting one rib-cartilage level to improve visibility and facilitate the microvascular anastomosis in these difficult cases. The flap is placed in the same pocket where the previous implant was; no new pocket or change of plane is advocated by our team.

After dividing the pedicle, the flap is transferred to the thorax for anastomosis to the internal mammary vessels with 9-0 interrupted nylon sutures under surgical microscope augmentation. The flap is deepithelialized, and a skin paddle is left in place to monitor the flap, usually where the implant was exposed to make up for the lost tissue in that area. The flap is inserted in the pocket and the wound closed in two layers with 3-0 Monocryl on top of a suction drain.

**Fig. 1:**
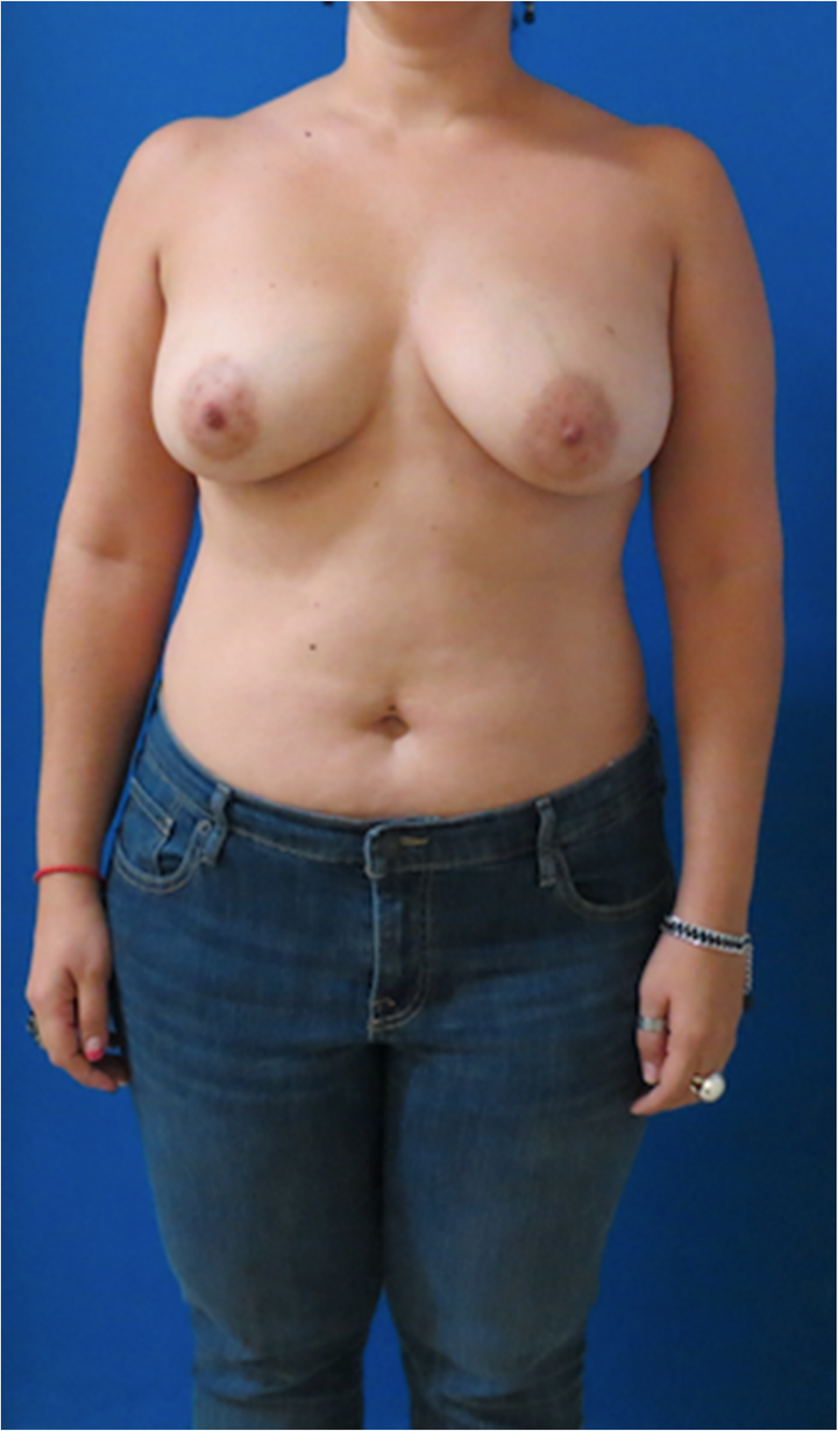
Case 1. Preoperative view before right prophylactic nipple-sparing mastectomy and left therapeutic nipple-sparing mastectomy with immediate bilateral implant-based reconstruction

**Fig. 2:**
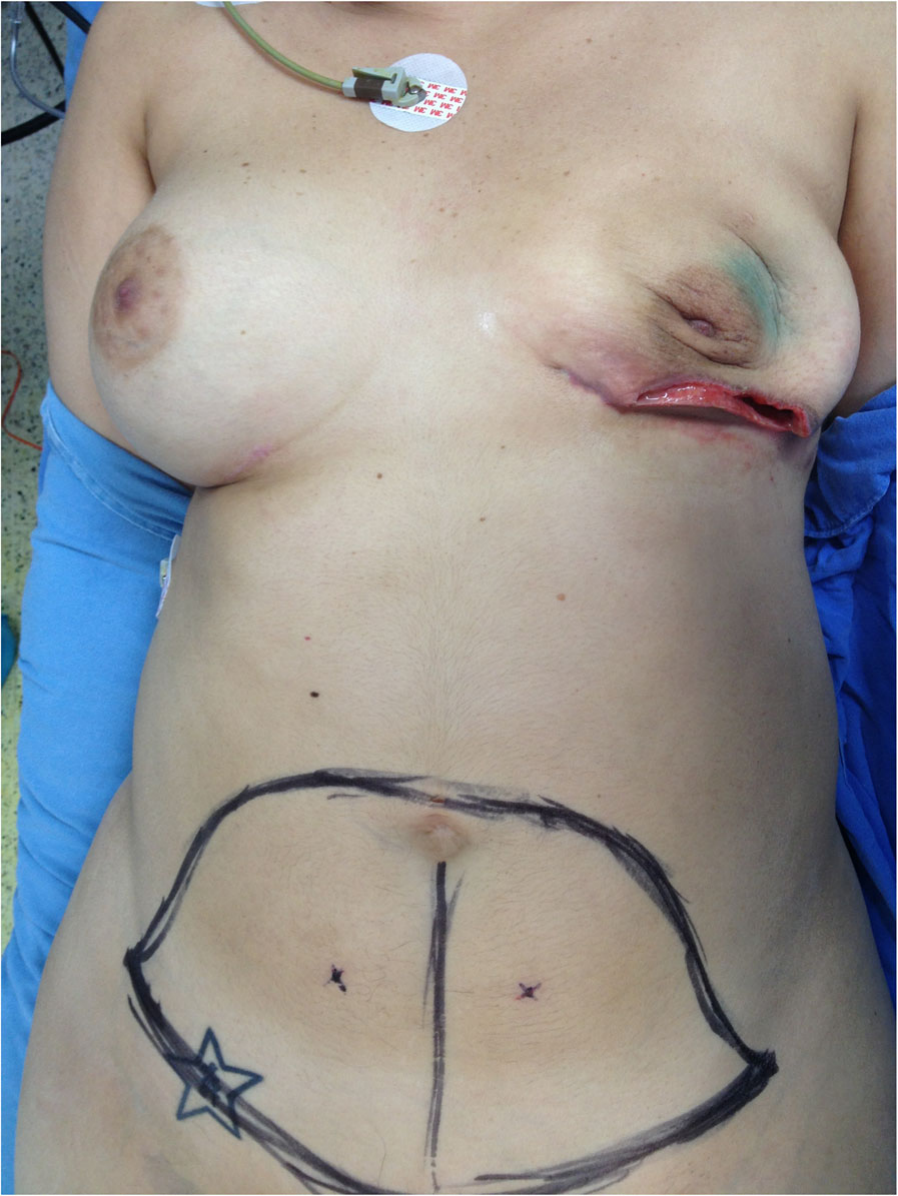
Case 1. After failed reconstruction with implant exposure on the left breast, the patient was recruited in our study. The left implant was removed. Trans-operative view before DIEP flap harvest

**Fig. 3:**
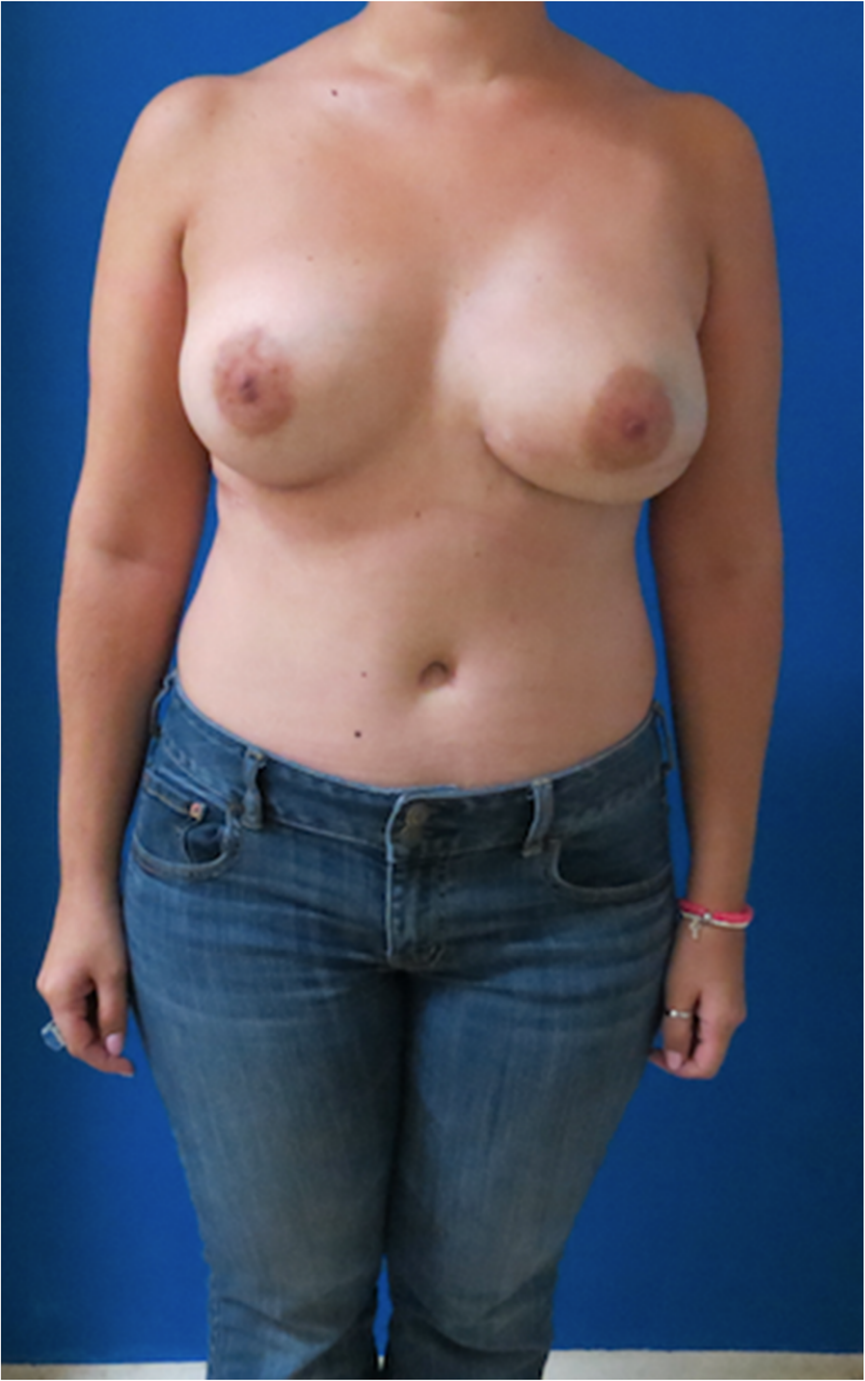
Case 1. Postoperative view after 8 weeks of single-stage implant removal and replacement with DIEP flap

**Fig. 4:**
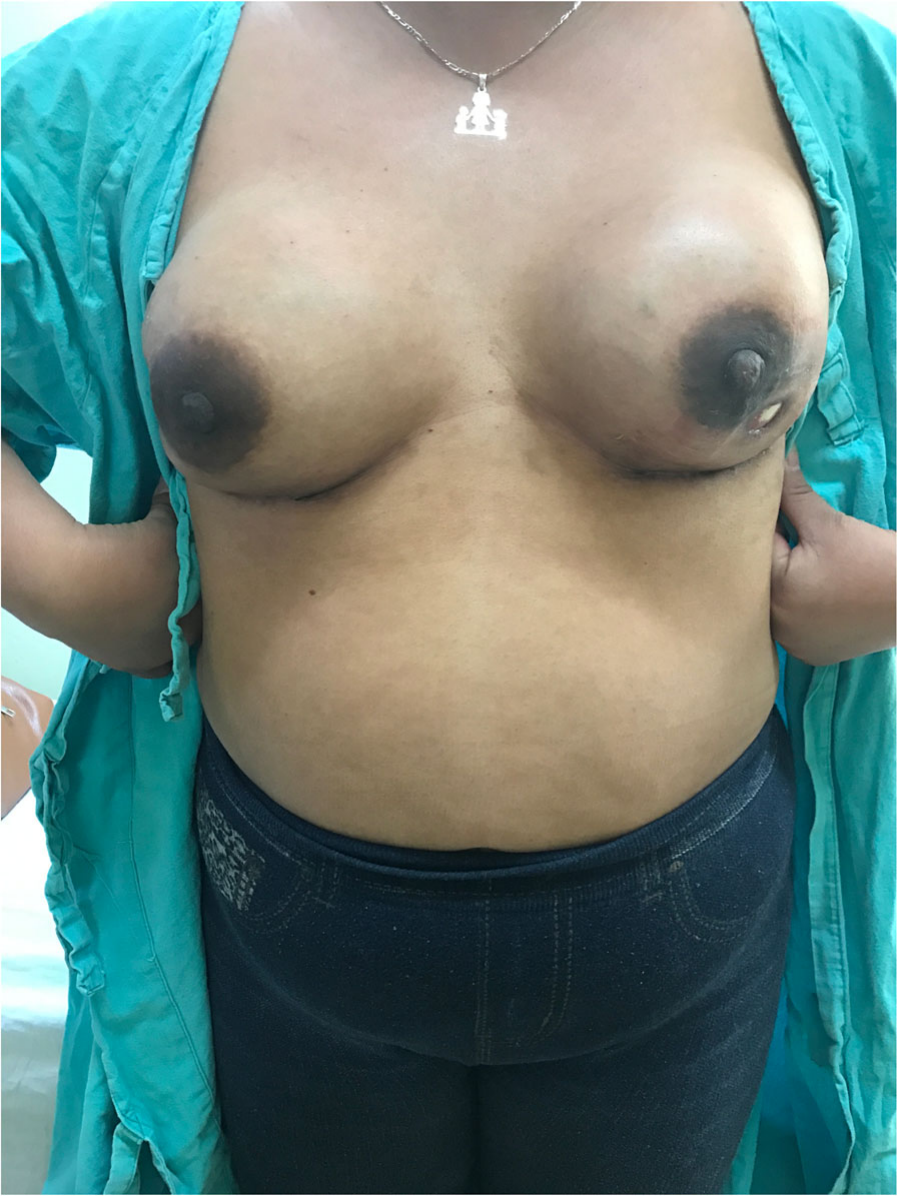
Case 2. Forty-five years old female after right nipple sparing therapeutic mastectomy and left prophylactic nipple-sparing mastectomy and immediate bilateral reconstruction with polyurethane round implants. Exposure of both polyurethane implants 5 weeks after the surgery

**Fig. 5:**
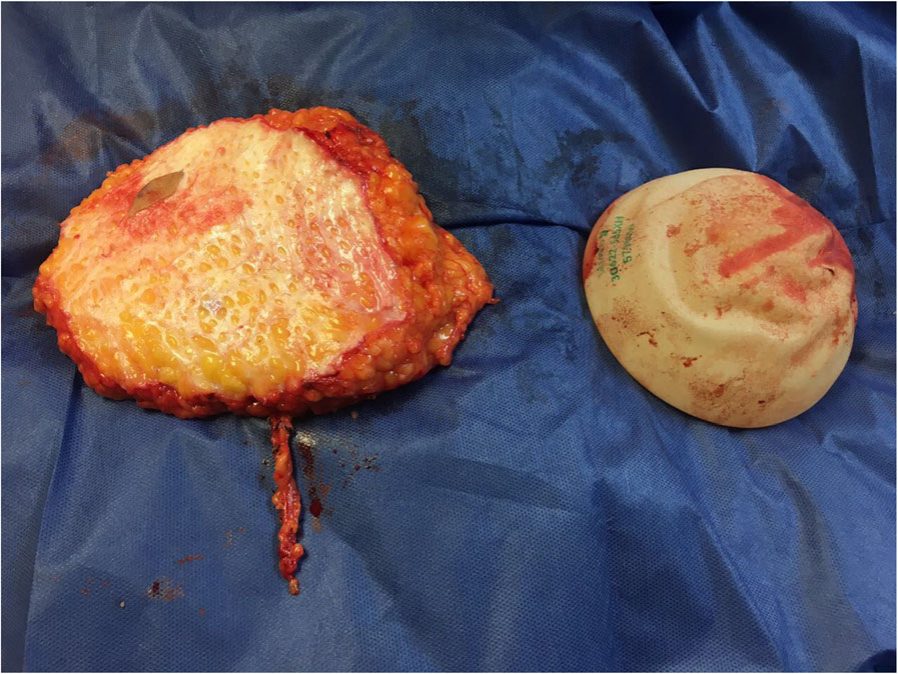
Removed implant and deepithelialized left DIEP flap

**Fig. 6:**
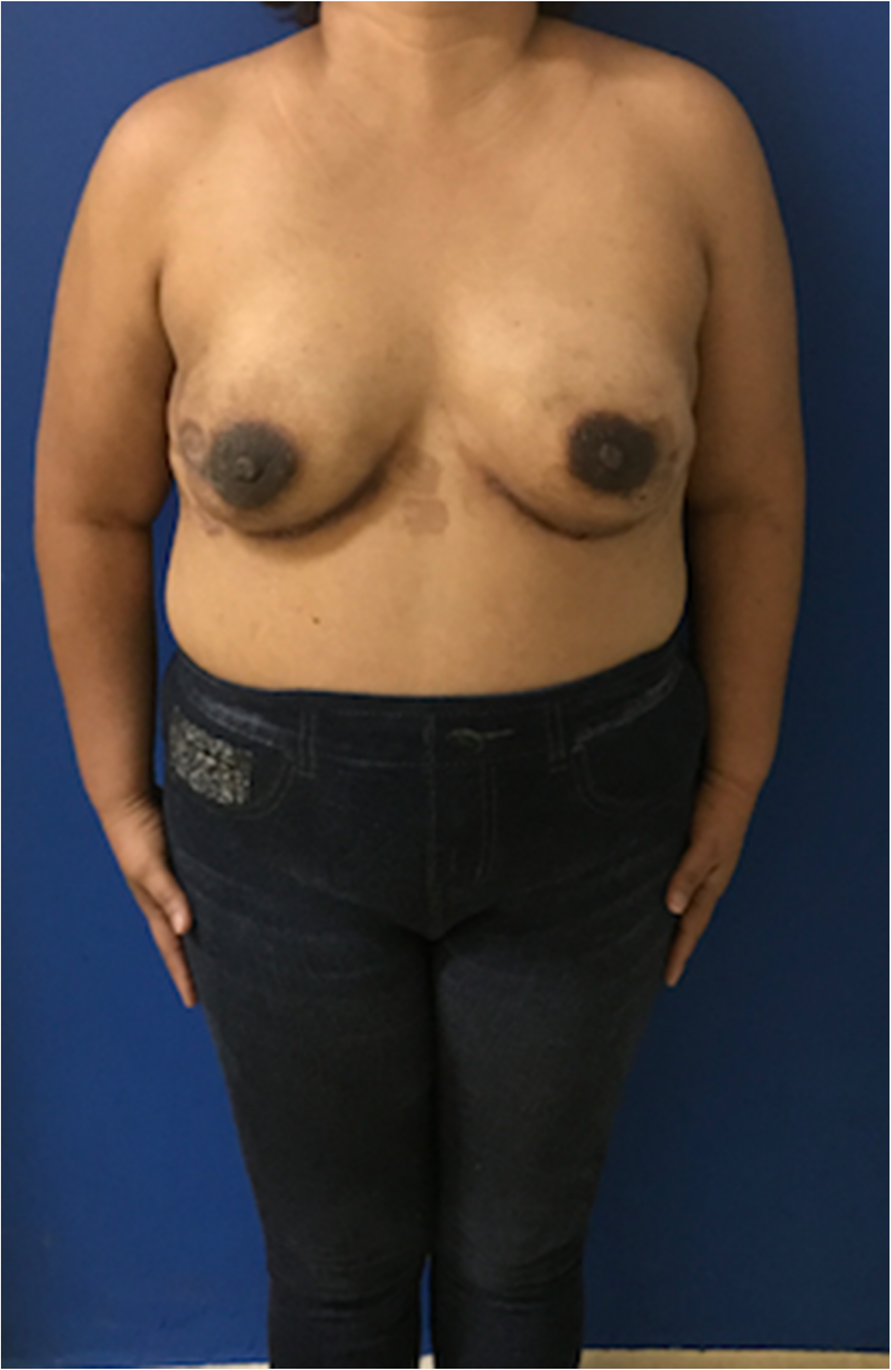
Case 2. Postoperative view after single-stage salvage reconstruction with bilateral implant replacement with bilateral de-epithelialized DIEP flap

The rectus sheath is closed with no tension, and the abdominal flap is advanced and closed in three layers over suction drains (Figs. 1, 2, 3, 4, 5, and 6).

## Results

From June 2012 through May 2017, 140 DIEP flaps were performed for breast reconstruction in the Department of Microsurgery in our institution. Eight of these patients were included in our study as they were previously treated with nipple-sparing mastectomy and immediate reconstruction with implant and developed partial skin necrosis and implant exposure. Two of these patients suffered bilateral implant exposure. Immediate deepithelialized DIEP flap was performed in all patients. The two patients with bilateral implant exposure underwent tertiary reconstruction with immediate bilateral DIEP flaps.

Patients’ mean age was 45 years old, (42–50 years; SD = 3.30), and the average implant volume removed was 463 cm^3^ (410–525 cm^3^; SD = 47.14 cm^3^). The mean time from implant exposure to tertiary DIEP flap-based reconstruction was 8 days (7–14 days). Exposed implants were seven silicones, two polyurethanes, and one saline. All patients were referred from different institutions, so we ignored the frequency with which they use each kind of implant to learn the rate of failure according to the implant type. Mean hospital stay was 6 days after surgery.

Three of the reconstructed breasts (30%) presented seroma that needed re-exploration and placement of a new drain 5, 7, and 14 days after surgery, respectively. Of these cases, one had had silicone, one saline, and one polyurethane implants. No partial or total flap necrosis or infection was present in these patients.

## Discussion.

Nipple-sparing mastectomy and immediate implant-based reconstruction is increasingly accepted as a desirable option for patients with breast cancer or hereditary risk of breast cancer. The oncological safety of the procedure has been well demonstrated [7, 28]. However, ensuring total resection of glandular tissue might lead to extremely thin skin envelopes with compromised vasculature. Skin flap ischemia is reported to happen in 5 to 15% of cases, and short-term complication rate is about 33 to 55% with implant loss in 8 to 18% of patients [7, 14, 29, 30]. In our study, all eight patients presented nipple-areola or skin necrosis and implant exposure that warranted prosthesis explantation.

Our technique for flap harvest does not differ from that previously described [17, 31–35]; however, flap inset varies in some aspects. First, if the third costal cartilage is far away from the skin defect, the fourth rib cartilage is preferred. In case of peri-areolar exposure, the third cartilage is used. Next, our experience is that capsulotomy is sufficient to provide chest flap compliance, allowing to create enough space to fit in the DIEP flap. Also, avoiding capsulectomy which, for us, has been unnecessary decreases bleeding and reduces surgery time. Fear about infection when using the same pocket is understandable, but it seems that the new, well-vascularized tissue and postoperative antibiotic coverage are enough to avoid it as we had no case of infection after surgery.

Changing the plane of dissection by creating a new pocket as suggested by Bramhall on his recent publication [36] would also lead to increased bleeding and time in the operating room. On the downside, three patients (30%) in our study presented seroma which needed draining. It is possible that our capsulotomy-only approach could be related to this complication, but further comparative studies are needed to objectively support this theory. All three seroma cases were resolved by placing a new drain without further complications.

Positioning the skin monitor of the DIEP flap where the implant was exposed serves two purposes: adequate flap monitoring and to avoid further breast deformity by replacing missing tissue lost due to skin necrosis. The rest of the flap needs only to be deepithelialized as it is proven that the dermis plays a significant role in enhancing the overall DIEP flap perfusion through the preservation of indirect linking vessels in the subdermal plexus [37].

Tertiary breast reconstruction after implant failure or capsular contracture has been widely discussed [17, 20, 38, 39]. However, to our knowledge, there is only one recent publication in English literature addressing the possibility of one-stage implant removal and immediate autologous reconstruction [36]. This technique addresses some problems found in delayed tertiary reconstruction such as skin retraction and added fibrosis, not to mention the cost and risks associated with an additional surgery. We believe that, on the psychological standpoint, the immediate implant replacement with DIEP flap is also beneficial to the patient just as the impact of immediate breast reconstruction is better than that of the delayed one [40, 41].

## Conclusion

Nipple-sparing mastectomy and immediate implant-based reconstruction is an oncologically safe technique. However, it has a high rate of complications that could necessitate implant removal. Immediate tertiary DIEP flap reconstruction with our technique replaces the exposed implant with low risk of infection (no cases in this study) and approximately 30% rate of seroma that can be resolved by appropriate draining with no further complications. It results in a soft and natural final breast shape.
